# Co-designing strategies to enhance engagement in telehealth exercise classes among cancer survivors: a participatory study applying identity leadership theory and introducing the SIGN framework

**DOI:** 10.1007/s11764-026-01966-x

**Published:** 2026-02-06

**Authors:** Camille E. Short, Mark Stevens, Tegan Cruwys, Sarah Stratulate, Tamara Jones

**Affiliations:** 1 Melbourne Centre for Behaviour Change, Melbourne School of Psychological Sciences, University of Melbourne, Melbourne, Australia; 2 CanRex Research Group, Department of Physiotherapy, Melbourne School of Health Sciences, University of Melbourne, Melbourne, Australia; 3 School of Medicine and Psychology, The Australian National University, Canberra, Australia

**Keywords:** Exercise oncology, Consumer involvement, Social identity leadership, Behaviour change, Online exercise programmes

## Abstract

**Purpose:**

To identify and evaluate consumer-informed strategies that online exercise leaders can use to enhance engagement in online exercise classes for people living with cancer, using social identity leadership theory as a guiding framework.

**Method:**

We used a participatory design involving adults with a history of blood cancer. Fourteen participants attended online co-design workshops to generate strategies that online exercise leaders could use to demonstrate their engagement in the four aspects of social identity leadership. Twenty-nine unique strategies were identified and thematically grouped using both social identity leadership theory and inductive analysis. These strategies were then rated by 18 participants on their potential to enhance commitment and appeal (0–100 scales).

**Results:**

Strategies were categorised into four domains-The SIGN Framework: Social support and connection, Individualisation and inclusion, Goals, monitoring and feedback, and Novelty. The highest-rated strategies primarily fell within the *Individualisation and inclusion* and *Goals, monitoring and feedbac*k domains, including tailoring exercises, setting personalised goals, adapting sessions based on well-being, and providing regular feedback. These behaviours aligned with the social identity leadership process *Identity advancement,* whereby leaders act in the group’s interest by supporting group members’ needs and progress. Qualitative findings highlighted that leaders who validate individuals’ needs and preferences strengthen their sense of inclusion and group belonging. Strategies involving *Social support and connection* (e.g., establishing group rules) and *Novelty* (e.g., weekly themes) were appreciated by some participants but received more mixed ratings.

**Conclusion:**

Online exercise leaders who act in the group’s interest and offer personalised support may enhance engagement and group identification among cancer survivors. The SIGN framework offers a practical guide for translating social identity leadership into online exercise settings.

**Implications for cancer survivors:**

Supporting exercise leaders to apply social identity leadership strategies may increase motivation, connection, and long-term participation in online programs for cancer survivors.

**Supplementary Information:**

The online version contains supplementary material available at https://doi.org/10.1007/s11764-026-01966-x.

## Introduction

Exercise is an effective intervention for improving cancer survivors’ physical and mental health, across different cancer types and stages of disease [1]. Substantial RCT evidence demonstrates that its benefits include mitigating treatment side-effects, decreasing disease risk, and reducing anxiety and depression symptoms [1–3]. However, providing equitable access to structured exercise oncology programs remains a significant challenge [4]. Telehealth programs have emerged as a promising solution [5, 6], and the COVID-19 pandemic has accelerated their integration into routine care [7]. However, while such programs provide a unique opportunity to expand support for cancer survivors, their effectiveness depends on whether they can meet all participants’ needs.

A key concern is the reported lack of social connection in telehealth exercise programs. Cancer survivors often describe online exercise classes as more isolating than those delivered face-to-face [8, 9]. This is particularly problematic because social connection plays a key role in promoting physical activity adherence [10–12], building self-efficacy [13], and supporting mental health [14]. Despite increasing adoption of telehealth formats, no guidelines provide recommendations for how exercise professionals (i.e., exercise *leaders*) who facilitate online exercise classes can foster positive group dynamics and connection in these exercise settings. Existing recommendations from professional associations and technology providers tend to focus on technical and clinical considerations [15–17], but there has been limited integration of psychosocial theory or consumer perspectives.

One theoretical framework that could be used to inform such guidelines is the social identity approach to leadership. The social identity approach is a dominant framework in social psychology for understanding how social groups impact people’s psychology and behaviours [18–20]. This framework proposes that people define themselves in terms of both their personal identity (as “I” and “me”) and their various *social identities* (i.e., as members of the social groups to which they belong; “we” and “us”). It further proposes that categorising oneself in terms of a social identity that you share with others (e.g., as a member of a particular online exercise class) lays the foundation for various forms of group behaviour, including social influence [18]. The social identity approach to leadership [21] builds on this, proposing that leadership is a *process of social influence,* and that a leader’s capacity to exert influence, and foster positive group member outcomes more holistically, rests on the extent to which they attend to, and help strengthen, group members’ social identity as a member of the focal group. It further argues that leaders can achieve this by (a) *representing* the group’s identity (identity prototypicality), (b) *advancing* the group’s identity and interests (identity advancement), (c) playing an active role in *creating*, and shaping the content of the group’s identity (identity entrepreneurship), and (d) helping *embed* the group’s identity in reality by providing practical activities that enable members to ‘live out’ their shared identity (identity impresarioship).

In exercise settings, research has shown that social identity leadership, and the stronger sense of social group belonging this engenders, is associated with greater attendance, engagement, and enjoyment [10–12, 21]. While most studies have been conducted within in-person exercise or sport settings (see [22] for a review), one recent study provides preliminary support for benefits of social identity leadership in online exercise settings. In a secondary analysis of RCT data, Reis et al. [23] pooled data from telehealth exercise class conditions (group and personal) and found that older adults who identified more strongly as member of their respective exercise programs demonstrated greater program adherence over 12 weeks. However, while recent research has begun to focus on identifying effective social identity leadership behaviours in face-to-face exercise classes [24], research has not identified the specific actions that leaders can take to foster social identification and engagement in telehealth settings, where connection can be more difficult to cultivate.

Addressing this knowledge gap is crucial for translating social identity leadership theory into practice. Doing so would also address the need for theory-informed evidence-based strategies to facilitate exercise professionals’ efforts to create telehealth environments that not only deliver safe and effective exercise, but also foster connection, motivation, and belonging. Furthermore, involving consumers in this process is essential to ensure the resulting recommendations are meaningful, acceptable, and likely to foster engagement [25, 26]. This project aimed to identify concrete actions that exercise leaders could take to demonstrate their social identity leadership within telehealth delivered exercise classes, drawing on the lived experience of blood cancer patients. Further, we aimed to gain insight into the perceived potential of the strategies identified to enhance commitment to and appeal of telehealth exercise classes. Although this work was conducted with blood cancer patients, who have distinct treatment experiences and support needs [27], the insights generated, including the development of the SIGN framework, are intended to provide a foundation for improving telehealth exercise delivery across diverse cancer populations, many of whom share similar challenges and barriers to exercise and connection in online settings [8, 28]

## Method

### Design

This participatory research study used co-design workshops to identify consumer and theory-informed strategies and a follow-up survey to assess strategy appeal and the extent to which participants felt the strategies would enhance their commitment to telehealth classes. Ethics approval was obtained from the University of Melbourne Human Research STEMM 3 Ethics Committee (Project number: 2023–26776-43,608–4).

### Participants and recruitment

The study was conducted as part of a larger program of work focused on the optimisation of a hybrid exercise service within a large tertiary hospital for people with blood cancer [29] and was therefore limited to those with a history of blood cancer. Participants were drawn from a sample of people (N = 80) who had previously participated in exercise-related survey studies conducted by the research team. Of these, 62 were from a survey conducted with patients at Peter MacCallum Cancer Centre who had completed a bone marrow transplant within the last three years, and 18 were from a national community-recruited sample who were eligible if they were within five years post diagnosis of blood cancer. We aimed to recruit a maximum of 30 participants to take part in co-design (including the follow-up survey), considering both data adequacy [30] and optimal numbers for co-design workshops in the time available [31]. We invited participants in batches, expecting a consent rate between 40–50% based on our prior research.

### Procedure

Potential participants were emailed a brief introduction to the study and provided with a link to the Plain Language Statement and Consent Form using REDCap (Research Electronic Data Capture) tools hosted at The University of Melbourne [32]. Consenting participants were invited to complete a poll indicating their availability for an online workshop. Workshops were planned for when at least four people could attend and took place in September 2023. Participants were informed that if they could not attend any of the workshops, they would still have an opportunity to provide input via a survey, which was conducted in October 2023. All consenting participants were emailed a link to the survey via Qualtrics within one month of the first workshop.

### Data collection and analysis

#### Workshops

**Fig. 1:**
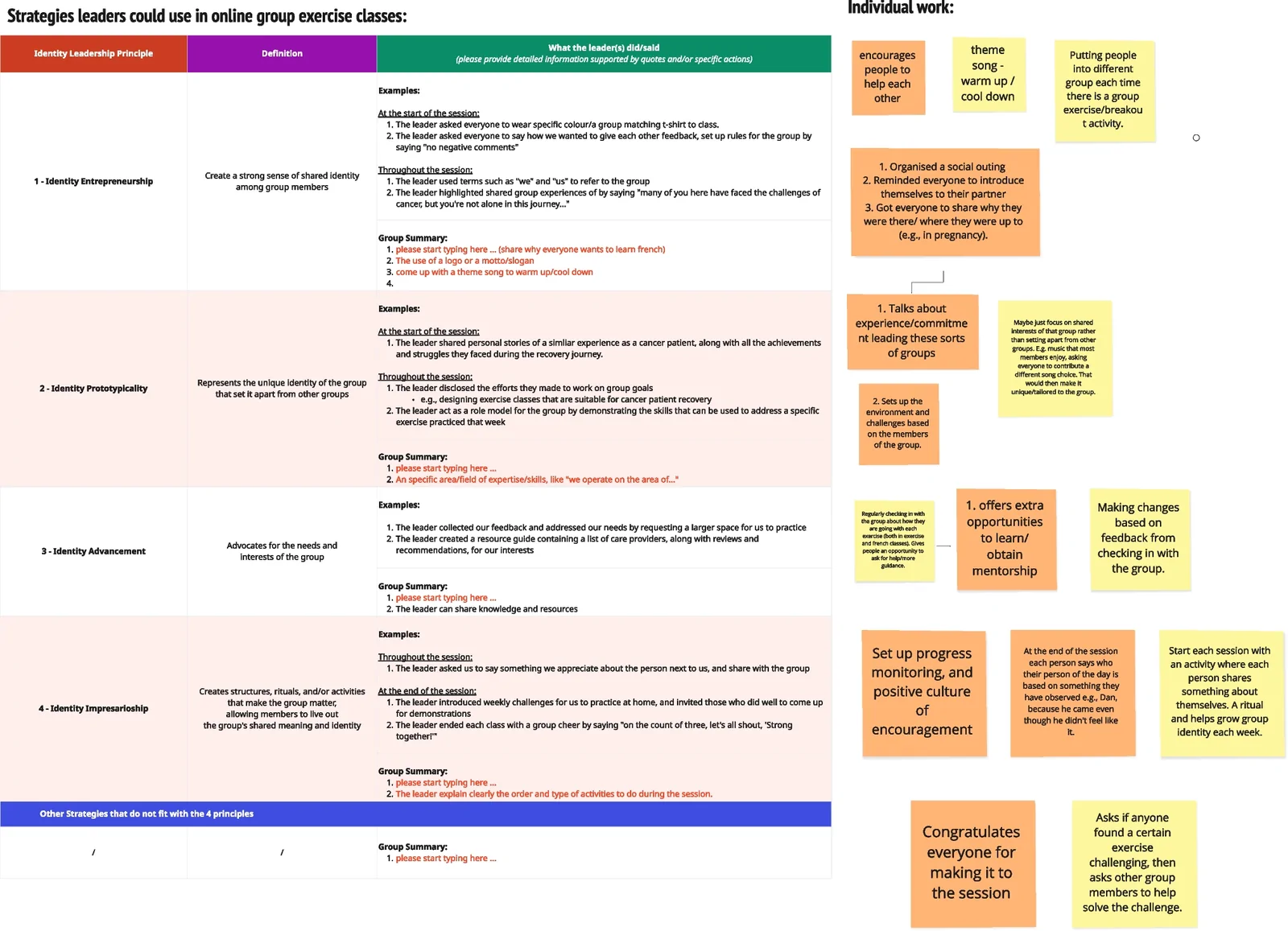
Miro board activity used during intervention mapping task

Two workshops were conducted online and recorded with permission using Zoom (Windows Version 6.1.11). Each workshop was 2.5 h in duration and facilitated by the research team. The workshops began with social introductions and establishing rules to make sure everyone felt safe and able to engage. A word-cloud activity was used as a warm-up to get participants thinking about and sharing their exercise class experiences in a non-threatening way. Participants were asked to input three words into the word-cloud that described their experiences, and all words were displayed for everyone to see. After brief discussion, participants were directed into smaller break-out rooms and given points to discuss around the groups they had been involved in, what behaviours and attributes they value in group leaders, and how the behaviours and attributes they identified made them feel. For this activity, to help facilitate discussion, participants were instructed to draw on *any* group activities they had been involved in, including non-exercise activities or group activities conducted in person. Each break-out group reported back to the entire group. After a short break, the research team then provided an overview of social identity leadership theory. Participants then returned to the breakout rooms where members of the research team facilitated an intervention mapping task using Miro (see Fig. 1). Here, participants were asked to identify strategies relating to the different dimensions of social identity leadership that they thought could be used specifically in an online exercise class setting. Examples were given to help participants understand the concept. The facilitators shared the Miro board on their screen for all participants to see and acted as a scribe, including helping to link suggested strategies to the framework where possible.

#### Strategy selection and categorisation

After the second workshop (21st September 2023), workshop recordings were reviewed to collate all strategies suggested by participants. Each strategy was listed in an Excel spreadsheet to enable them to be grouped. A concise list of 29 strategies was generated by merging conceptually similar strategies together. For example, if multiple people suggested tracking goals but via different methods (e.g., via leader boards, spreadsheets etc.) these were merged into the one strategy with multiple examples used to ensure all ideas were captured. Recruitment was closed at this point, as the research team judged that the dataset was sufficiently rich and diverse to support robust analysis, and that the added value of further data collection would not outweigh the time and resource costs [33].

The strategies were then coded in Excel using an inductive approach prior to the survey study. Inductive coding was conducted by behaviour change experts (CS, TJ) using a data-driven, atheoretical approach grounded in behaviour change principles (e.g., [34, 35]). This approach also aimed to frame the strategies in language and categories that would be accessible and meaningful to a lay audience, supporting the practical translation of consumer-identified strategies into online exercise contexts. The inductive coding process produced a set of broad themes, which were used to structure the survey. The themes are presented in the Results section. Deductive coding using tables in Microsoft Word was then undertaken by social identity leadership experts (MS and TC) to determine how the identified strategies and inductive themes mapped onto the four social identity leadership domains: identity prototypicality, advancement, entrepreneurship, and impresarioship.

#### Survey

Participants were subsequently presented with the 29 identified strategies and asked to rate each strategy in terms of how much it (1) would help them feel *committed* to an online exercise group and (2) *appealed* to them on a scale from 0 (not at all) to 100 (to a great extent). To break up the task for participants in a lay-friendly way, strategies were presented in blocks under the four (inductive) thematic headings.

Demographic and health information for each participant was available from the prior survey studies and was not reassessed as part of this study. Descriptive statistics were calculated for all study variables. Quotes relating to participants’ lived experience of the strategies were also extracted and are presented to embed consumer voices in the results.

## Results

### Participants

All 80 people on our contact list were invited to participate. Twenty-three participants consented to participate in the study, and 18 participated in the workshop (n = 14) and/or survey (N = 17). Participants (N = 18; 56% Female) had a mean age of 61.6 years (SD 7.7) and an average BMI of 27.2 (SD = 6.1). Most participants were diagnosed with myeloma (61%), living with a partner (89%), had a university degree (61%) and lived in regional areas (61%). Demographics for those who completed the survey and the workshops were similar, except more male participants completed the survey only (workshops 33% male participants; survey 47% male participants). Further demographics are presented in Supplementary Table 1.

### Overview of consumer-identified strategies

The 29 consumer-identified strategies mapped to three of the four social identity leadership dimensions: advancement, entrepreneurship, and impresarioship. Notably, no strategies were classified under identity prototypicality. Five strategies that were initially difficult to classify were later re-coded based on participant quotes that suggested they played a role in fostering group belonging (see Supplementary Table 2 for initial coding, quotes considered, and changes made).

**Fig. 2:**
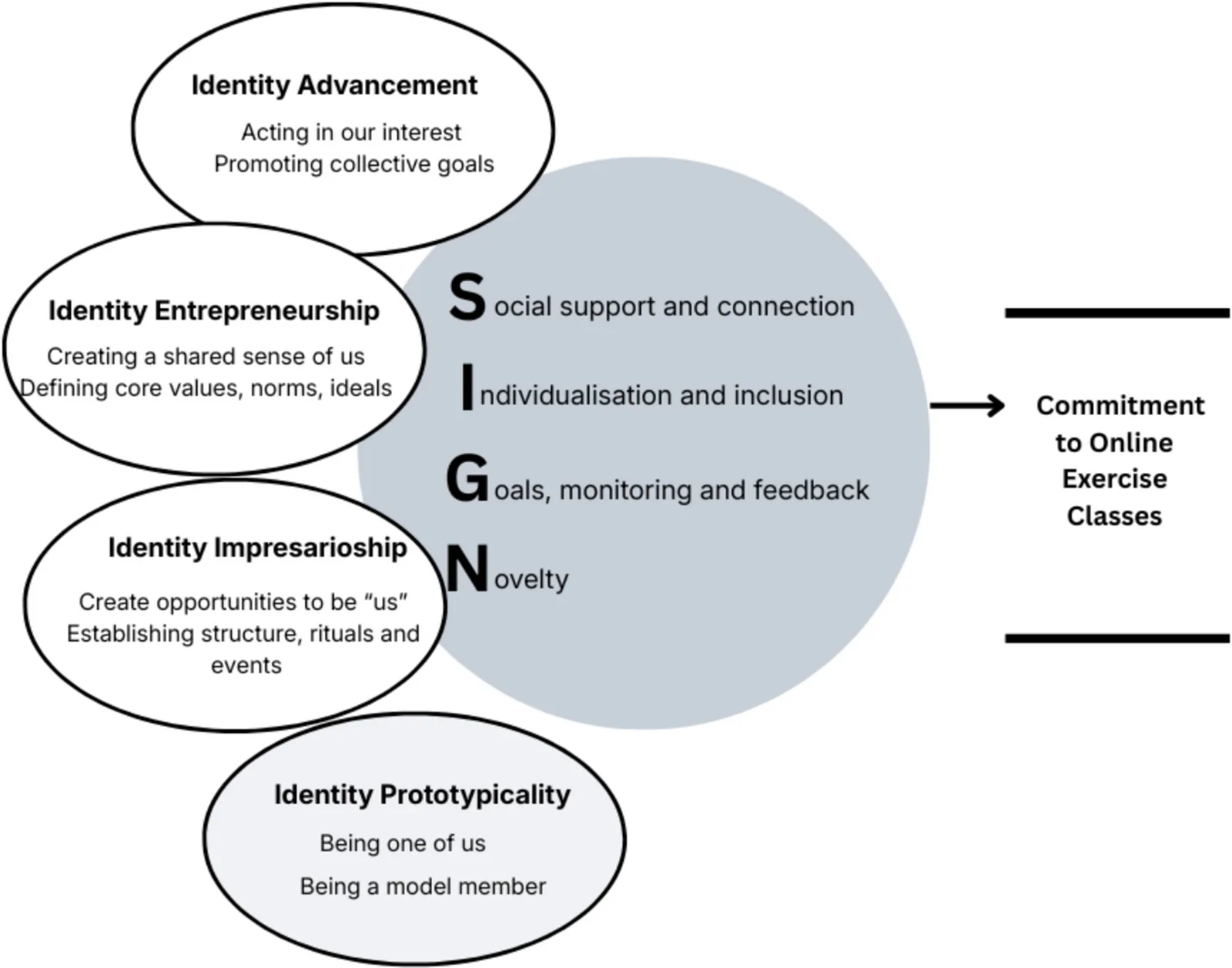
Adapted from [36]: The 29 identified strategies map onto three of the four identity leadership domains (excluding identity prototypicality). The SIGN framework supports the practical translation of these strategies by serving as user-friendly mnemonic aligned with existing behaviour change approaches within exercise oncology. By utilising SIGN-strategies linked to social identity leadership processes, exercise leaders can enhance commitment to online exercise classes

Based on the inductive thematic analysis, strategies were grouped into four overarching themes—*Social support and connection; Individualisation and inclusion; Goals, monitoring and feedback*; and *Novelty*—which we collectively referred to as the SIGN framework (see Fig. 2).

Across the 29 strategies, ratings of commitment and appeal were generally well aligned, with most strategies receiving similar ratings on both dimensions (within 5 points on average, on a scale ranging from 0–100). There were a number of small divergences, however, these were small in size and did not follow a consistent pattern across SIGN themes or identity leadership constructs. Descriptive statistics, including means and medians for participants’ ratings of all 29 strategies, as well as the alignment of each strategy with both frameworks are summarised in Table 1.

**Table 1:** Consumer-Identified Strategies to Enhance Engagement in Online Exercise Programs: Ratings of Perceived Commitment and Appeal

	Strategies	Identity leadership principle	The following strategies would help you feel committed to the online exercise group On a scale from 0 (not at all) to 100 (to a great extent)					The following strategies would **appeal to you.** On a scale from 0 (not at all) to 100 (to a great extent),				
			Mean	SD	Median	Min	Max	Mean	SD	Median	Min	Max
**SOCIAL SUPPORT AND CONNECTION**												
1	Build trust within the group to make people feel comfortable sharing their struggles or concerns towards doing the exercise (e.g., no judging)	Entrepreneurship	78.1	26.7	84.0	14.0	100.0	69.5	28.1	80.0	5.0	100.0
2	Set up clear and respectful group rules (e.g., taboo topics—particular topics that shouldn't be discussed within the group)	Entrepreneurship	77.6	30.0	90.5	10.0	100.0	76.1	30.4	84.0	10.0	100.0
3	Pair up people with similar fitness, treatment level and/or interests (e.g., buddy system)	Advancement	73.3	22.0	77.5	20.0	100.0	74.5	29.3	88.0	11.0	100.0
4	Create opportunity for people to interact outside of the class (e.g., discussion board, emails communication)	Impresarioship	69.9	28.5	75.0	6.0	100.0	64.2	32.5	68.5	4.0	100.0
5	Make time to talk during the class, allowing people to share their stories, thoughts, concerns, and experiences	Entrepreneurship	69.5	25.7	70.5	20.0	100.0	69.3	28.1	79.0	9.0	100.0
6	Recognize people's commitment to the class and provide group support as an encouragement for that commitment (e.g., cheer for each other’s accomplishments)	Advancement	67.5	25.0	73.0	19.0	100.0	64.6	32.4	72.0	5.0	100.0
7	Organize a group chat on social media (e.g., a Facebook group)	Impresarioship	64.8	29.6	74.5	4.0	100.0	64.3	33.2	71.5	4.0	100.0
8	Facilitate options to hang around immediately before or after the class (e.g., stay behind on zoom)	Impresarioship	63.4	30.4	72.5	5.0	100.0	63.3	34.5	69.5	4.0	100.0
**INDIVIDULISATION (AND INCLUSION)**												
9	Structure the exercise to match with individual's needs and physical circumstances	Advancement	91.5	9.2	95.0	75.0	100.0	88.2	15.2	94.0	55.0	100.0
10	Design the program based on group needs (e.g., always having a go slow option or easier alternative for each exercise)	Advancement	89.3	12.2	93.0	60.0	100.0	85.7	14.5	89.5	55.0	100.0
11	Conduct baseline and follow-up assessments (e.g., physical ability testing)	Advancement	88.2	15.5	97.0	51.0	100.0	82.5	22.7	90.5	26.0	100.0
12	Get to know how people are feeling on a particular day (e.g., traffic light system: red/yellow/green – well/soso/unwell) and adapt the exercise based on that	Advancement/Entrepreneurship	84.4	16.3	88.5	50.0	100.0	81.9	24.3	89.0	10.0	100.0
13	Get to know people in the group individually (e.g., their limitations, needs) before starting to work with them	Advancement	83.5	17.4	91.5	51.0	100.0	83.1	14.6	84.5	59.0	100.0
14	Deliver the class in short exercise segments, allowing people to have breaks and/or a shorter session if needed	Advancement	74.7	20.4	82.5	34.0	100.0	75.3	27.5	85.0	10.0	100.0
15	Do a survey prior to draw on the group's common grounds, understand their needs and interests	Advancement	70.9	23.7	78.5	15.0	100.0	68.4	29.1	76.0	10.0	100.0
16	Refer to the group using collective language (e.g., "the team", "we", and "us")	Entrepreneurship	60.3	29.3	62.5	10.0	100.0	55.6	37.3	60.0	6.0	100.0
	**GOALS (FEEDBACK & MONITORING)**											
17	Set up personalized goals for individuals based on each person's physical ability, fitness, and/or treatment level	Advancement	89.3	16.0	99.5	56.0	100.0	91.9	12.2	97.5	62.0	100.0
18	Monitor individual and group progress closely and step in when needed to give feedback	Advancement	83.0	16.9	84.0	49.0	100.0	85.3	15.5	89.0	43.0	100.0
19	Ask the group to share their individual short-term and/or long-term goals	Impresarioship	71.2	30.0	81.5	1.0	100.0	74.4	27.4	84.5	10.0	100.0
20	Work with the group to identify their aims/goals as a group	Advancement	70.5	28.0	82.5	15.0	100.0	65.8	32.9	75.5	7.0	100.0
21	Highlight the group's shared goal of achieving physical fitness, improved wellbeing, and a better quality of life	Entrepreneurship	69.7	31.6	79.0	5.0	100.0	76.0	27.1	84.5	11.0	100.0
22	Implement a structured tracking system (e.g., a scoreboard, spreadsheet, diagram) to measure individual and group progress	Impressarioship	67.9	24.5	71.0	25.0	100.0	73.4	25.7	81.0	15.0	100.0
	**NOVELTY**											
23	Put together a video for the group detailing the exercise and show how to do the exercise correctly	Advancement	87.7	19.5	92.5	22.0	100.0	81.3	26.0	87.0	12.0	100.0
24	Introduce weekly themes based on groups interests (e.g., grand-final week, managing fatigue)	Impresarioship	65.9	29.2	71.0	10.0	100.0	58.2	31.1	59.0	1.0	100.0
25	Introduce motivational word of the week for the group to reflect on (e.g., strength, hope, peace, mindfulness)	Impresarioship	62.9	30.9	65.5	1.0	100.0	64.3	32.5	72.5	1.0	100.0
26	Come up with a group name for the class reflecting on the group aim (i.e., what the class is for)	Entrepreneurship	60.0	28.5	62.0	10.0	100.0	57.8	37.5	58.0	7.0	100.0
27	Involve the group in music selection for class warm-ups and cool-downs	Impresarioship	58.7	24.3	61.0	18.0	93.0	54.5	29.3	56.0	9.0	100.0
28	Introduce weekly challenges (e.g., take a photo of a bird while out for a walk)	Impresarioship	57.6	29.5	56.5	1.0	95.0	60.8	29.5	64.0	1.0	100.0
29	Put up slides or content centred around the group that are visually relevant to the group aim or name	Impressarioship	55.1	31.9	60.5	4.0	100.0	54.7	33.6	59.0	1.0	100.0

Across all strategies, eight received particularly high ratings, with both mean and median scores above 80 on perceived potential to increase class commitment and appeal. These strategies primarily aligned with the *Individualisation and inclusion* theme and included tailoring exercises to individual capabilities, setting personalised goals, and conducting assessments to inform program design. One strategy fell under *Goals, monitoring and feedback*, while another aligned with the *Novelty* theme. Despite spanning different SIGN domains, in the deductive coding process all eight were classified as identity advancement – that is, they shared a common focus on supporting and advancing the interests of the group and its individual members. Importantly, participants described how these personalised forms of support, such as leaders recognising their needs, adapting exercises, or checking in made them feel seen, valued, and included within the group. Illustrative participant quotes that reflect this dynamic are presented throughout the SIGN sections below.

### The SIGN framework

#### Social support and connection

Participants highlighted the importance of social connection, both with the exercise leader and among fellow class members. While the virtual format differed from face-to-face delivery, many participants described forming meaningful relationships through online sessions. Leaders who were enthusiastic, attentive, and genuinely interested in participants’ well-being were viewed as motivating and effective. One participant noted, “You need someone who actually wants you to take part in their class… you want someone that actually wants to be there… not someone who is there under sufferance because they have to be there” (ID3, Female, 56 years). Participants also valued opportunities for informal interaction during class time, where they could share tips, personal experiences, or encouragement with others. For example, one described, “The time when we got to talk to each other was during the actual exercises… Someone would comment, ‘Have you tried this?’ or ‘Have you tried that?’… everyone got the benefit of that suggestion” (ID15, Male, 69 years).

Eight strategies were identified within this theme (see Table 1). Three strategies focused on leaders promoting group cohesion and a sense of what it means to be a group member: building trust (strategy 1), establishing clear and respectful group rules (strategy 2), and recognising commitment (strategy 6). Five were oriented toward facilitating social interaction within or around class sessions: leaders making time for peer sharing (strategy 5), setting up group chats on social media (strategy 7) or other forums (strategy 4), encouraging pre or post-class conversations (strategy 8), and introducing a buddy system (strategy 3). These strategies mapped to three social identity leadership components: identity entrepreneurship (e.g., setting norms and expectations to promote openness), identity advancement (e.g., acknowledging individual contributions and encouraging peer support), and identity impresarioship (e.g., using tools and structures to support interaction outside of structured sessions).

Quantitative ratings suggested moderate to strong support for these strategies, with all mean and median scores exceeding 60 for both perceived commitment to the class and appeal. The highest-rated strategies for commitment were setting clear and respectful group rules (strategy 2) and building trust within the group (strategy 1), both of which were examples of identity entrepreneurship. The buddy system strategy (strategy 3) was also highly rated and received the highest median appeal rating (88.0).

Qualitative data provided evidence of how leaders engaging in these strategies laid the foundation for downstream positive outcomes for class members. One participant explained, “I sent an email to the other participants… I said my name, that I’m part of the class… if anyone wants to catch up for coffee—and that has happened. I caught up with people that I only ever saw on the screen” (ID15, Male, 69 years). Further speaking to psychosocial benefits another participant noted: “I think it is genuinely part of the healing process that we forget about… being able to share our concerns, how we feel and our experiences” (ID9, Male, 69 years).

#### Individualisation and inclusion

Participants described individualisation as a defining feature of effective online exercise leadership. Leaders who adapted sessions to individual capabilities, asked about personal needs, and demonstrated responsiveness were perceived as caring, competent, and inclusive. These personalised interactions were not only seen as practical accommodations, but as relational acts that deepened participants’ sense of connection to the group. One participant reflected, “There were times when I felt ill and could hardly walk down the hallway… so trying to do an exercise class was beyond me. So it’s just that understanding of what treatments you are undergoing or what you have undergone that may vary your capability… It makes you feel connected” (ID1, Female, 67 years).

Eight strategies were identified within this theme. Several focused on leaders tailoring exercise delivery to individual needs (e.g., structuring sessions with built-in rest, offering adaptations for different physical capacities), while others emphasised the importance of leaders knowing the group (e.g., conducting assessments, using surveys, or checking in regularly). These strategies were primarily coded as identity advancement, as they reflected leader behaviours oriented toward supporting group members’ progress and wellbeing. As noted above, the advancement-related strategies had high levels of support for both commitment and appeal. One strategy in this cluster—*referring to the group using collective language* (e.g., “we,” “us,” or “the team”; strategy 16)—was aligned with identity entrepreneurship, as it helped define and reinforce a shared group identity (see also [37, 38]). While the effects of this strategy were not explicitly discussed by participants, this strategy may help signal that all members are part of a collective, thereby complementing the more personalised strategies within this theme. This was one of the lowest rated strategies across all categories but notably still had moderate support (see Table 1).

Qualitative accounts strongly reinforced the relational importance of individualisation. Participants described how instructors’ efforts to learn their names, check in about wellbeing, and adapt exercises contributed not just to a better exercise experience but also to a stronger sense of inclusion. For instance, “If you didn’t want to do it that day… it was, ‘nice to see you back, have you been okay?’… It made you feel like you were part of the group” (ID2, Female, 58 years). Another noted, “She’d do the exercises in the circuit class but then if she’d thought that I wasn’t able to do them, she’d adapt a different exercise… I think that gave people more confidence to be able to work at their level… and each week you could see that you were improving” (ID4, Female, 68 years).

The act of recognising individuals was often experienced as deeply validating. One participant described, “Getting to know people individually… that can really emanate into the whole group, whereby the leader is able to cater to individual needs but also integrate those needs within the group” (ID4, Female, 68 years). Others echoed this, highlighting that being checked on, addressed by name, and provided with options made them feel “looked after” and motivated to continue participating: “She was always checking on us… if you can’t do such and such, do this alternative… It made it feel very warm… like you wanted to go to the next session” (ID2, Female, 58 years). By tailoring programs to individuals, leaders not only improved access to exercise but also reinforced a sense of belonging. As one participant summarised, “I’m important, I’m being listened to here, I’m being understood… that can really emanate into the whole group… so that they feel like they belong” (ID4, Female, 68 years).

#### Goals, monitoring and feedback

Participants described goal-setting, progress monitoring, and feedback as important leadership strategies that enhanced motivation, supported individual effort, and reinforced a shared sense of purpose within the group. These strategies were valued not only for their practical benefits, such as structuring participation and gauging improvement, but also for their symbolic role in demonstrating leader attentiveness and investment in participant outcomes. When delivered with care and responsiveness, goal- and feedback-oriented behaviours were interpreted as signals that the leader recognised and valued each member’s progress.

Both individual and collective goals were highlighted as meaningful. Individually tailored goals were seen as necessary to accommodate differences in physical ability, treatment stage, and motivation. As one participant explained, “It’s useful to have a measurement… you start small and work up. It shows you that you’re improving” (ID3, Female, 56 years). Others emphasised the importance of creating a shared group aim to foster cohesion and collective purpose. For example, one participant reflected, “It’s really important to form some goals that the group wants to achieve… it creates that cohesion, that we’re all working towards a single thing” (ID4, Female, 68 years).

Six strategies were included under this theme. One focused on individual processes (setting personalised goals; strategy 17), three focused on individuals and the group (monitoring progress, providing feedback, sharing personal goals; strategies 18, 19, 22), and two targeted collective goal-setting practices (working with the group to identify goals, and highlighting shared group goals; strategies 20, 21). One additional strategy-conducting baseline and follow-up assessments (strategy 11) was discussed earlier in the *Individualisation and inclusion* section, but also fits conceptually here, as it was often described as a way to measure progress and tailor feedback over time. This was one of the strategies that was originally coded as not being a clear example of identity leadership, until participant quotes were considered (see supplementary Table 2).

Strategies within this theme mapped across multiple identity leadership components. Identity advancement was most evident in strategies that involved collaborating with participants to establish individual or group goals and providing tailored feedback. These approaches reflected leaders’ commitment to supporting members’ growth. As one participant put it, “The teacher should be giving feedback… positive and constructive. It’s part of the leader’s responsibility to ensure there’s benefit to be had” (ID10, Male, 68 years).

Identity entrepreneurship was reflected in efforts to define a motivating and meaningful collective aim, for instance, highlighting that the group’s shared goal was to improve physical function, wellbeing, and quality of life. Articulating this overarching purpose was seen as helpful in shaping the group’s identity and reinforcing why members were participating together. One participant noted, “It’s about, okay, we’re all here because we want to get stronger, we want our bodies to work better for us, we want to be able to have better quality of life. So it’s looking at it from those kind of points of view rather than the point of view of ‘well, you have got cancer so you need to be in this class to you know, whatever” (ID4, Female, 68 years). This shift in framing, from illness-based necessity to shared, positive aspirations illustrate how identity entrepreneurship can support a more empowering group narrative.

Identity impresarioship was demonstrated in strategies that made progress and development more visible, such as asking members to share their goals, or using tools like spreadsheets and tracking systems to map performance over time. These practical approaches gave form to the group’s progress, embedding its purpose into everyday class activity.

#### Novelty

Participants described a range of novel strategies that enhanced enjoyment, emotional engagement, and connection within the online exercise environment. Many of these strategies introduced symbolic or thematic elements into the session structure (e.g., weekly themes, motivational words, or participant-selected music). As one participant recalled, “She’d say a motivational word that you were to think about… like ‘strength’ or ‘hope’… and we all went away and thought about that. It was a nice thing” (ID2, Female, 58). Another commented, “The themed exercises-they’ll get you out the door more than thinking about calories or fitness. It’s the music and atmosphere that gets you there” (ID18, Female, 51). These strategies reflected identity impresarioship, in which leaders use tools, rituals, and symbolic content to give form and meaning to group identity. One such strategy- creating a group name (strategy 26)- was aligned with identity entrepreneurship, as it helped define “who we are” and establish the group as a distinct and cohesive entity.

In contrast, the most strongly endorsed strategy in this category was the development of a tailored exercise video for the group (strategy 23), which would allow participants to safely engage with exercises outside of class. This approach, aligned with identity advancement, was seen as highly valuable. As one participant explained, “I still do the exercises I was shown, but I never knew if I was doing them right… A video made by the leader, just for our group, would have made a huge difference” (ID1, Female, 67).

Overall, seven strategies were included within this theme. While most novelty strategies received moderate ratings, there was greater variability in how strongly they resonated with participants. Those reflecting identity impresarioship and entrepreneurship, such as themed sessions, symbolic content, or group naming, were appreciated by some, but not consistently considered valuable. In contrast, the video strategy (identity advancement) was among the most highly rated of all behaviours, with a median commitment score of 92.5 and appeal score of 87.0.

## Discussion

This study identified 29 consumer-informed strategies to enhance engagement and commitment in online exercise programs for people living with cancer. These strategies mapped onto three of the four dimensions of social identity leadership theory-entrepreneurship, impresarioship, and advancement-with qualitative and quantitative data suggesting that strategies aligned with identity advancement were consistently most valued. More specifically, participants placed particular importance on leaders demonstrating care and responsiveness through strategies such as tailoring exercise to individual needs, setting personalised goals, and providing regular feedback. These behaviours were seen as effective for both fostering exercise adherence and reinforcing a sense of inclusion, value, and group belonging.

In interpreting these findings, it is useful to consider the pattern of ratings across the two quantitative dimensions of appeal and commitment. Differences between the two typically ranged only 5–10 points on a 0–100 scale, suggesting that participants’ affective reactions to a strategy and their expectations of its value on impacting their commitment were generally well aligned. This is consistent with perspectives in digital health research which position acceptability (analogous to appeal) as closely related, yet not identical, to engagement (outcome of commitment), and propose that acceptability can influence engagement behaviour [39]. Thus, practitioners should be mindful when selecting strategies that have large variations in appeal ratings, as strategies that people find unappealing may impact on engagement in classes.

Interestingly, no strategies were classified as an example of identity prototypicality, which refers to a leader being seen as a representative embodiment of the group’s identity. This is notable given the centrality of prototypicality in previous identity leadership research and strong evidence for its benefits [40, 41]. In this context, participants appeared to view leaders as external support figures rather than peers who symbolise “who we are.” This may reflect the dynamics of clinical or supportive care environments, where expectations of expertise and professionalism supersede peer similarity- particularly given that exercise participants are cancer survivors, often facing ongoing physical limitations, while leaders are typically fit, sometimes younger individuals without a history of cancer. It is also possible that responses would differ if participants were prompted to consider peer-led exercise leadership. Future work could explore whether perceptions of leader prototypicality shift over time, particularly as leaders demonstrate alignment with group norms such as care, empathy, and mutual support.

To organise the wide range of suggested strategies, we developed the SIGN framework, grouping the strategies into four practical domains: Social Support and Connection, Individualisation and Inclusion, Goals, Monitoring and Feedback, and Novelty. This framework offers a flexible, user-friendly structure for translating social identity leadership principles into practice. SIGN aligns closely with well-established behaviour change strategies, such as tailoring, social support, goal-setting, and feedback, which have been shown to enhance physical activity participation [42–47]. Importantly, however, SIGN also captures how these mechanisms can be delivered in ways that support not just behaviour change, but also social identity development. For example, helping participants track progress is not merely a motivational tool, it can also reinforce a sense of shared journey and collective investment in the group.

Given the breadth of identified strategies, it is neither necessary nor realistic for exercise leaders to implement all 29 strategies. There is no ‘one-size-fits-all’ approach that will guarantee positive outcomes; the most appropriate strategies will depend on leaders’ skills and preferences, the needs and values of group members, and the practical context in which classes are delivered. Instead, the SIGN framework and our preliminary data on commitment and appeal is intended to support informed selection by prompting leaders to consider a broad repertoire of options across four domains—Social support and connection, Individualisation (and inclusion), Goals (monitoring and feedback), and Novelty (SIGN). By drawing from each domain where feasible, practitioners can flexibly tailor their approach while ensuring that core psychosocial mechanisms underpinning engagement and group identification are addressed.

From a practical standpoint, the SIGN framework provides exercise professionals with a clear and actionable guide to support cancer patients’ engagement in online exercise classes. It encourages leaders to move beyond an exclusive focus on content delivery to consider how they can positively shape participants’ group experience by fostering emotional safety, tailoring to individual circumstances, reinforcing shared goals, and using creative elements to build group identity. While many of the highest-rated strategies fall under existing best practices in exercise oncology (e.g., personalised exercise programs; [48]), this study highlights their added value in cultivating social belonging, which is a critical but often under-acknowledged aspect of program engagement [8, 9, 23].

This sense of belonging may also reflect what social identity researchers describe as complementarity-that is, that group cohesion is sometimes best fostered not through group members being treated as uniform and interchangeable, but instead through recognition of each member’s unique value to the group. As theorised by Koudenburg et al. [49] (and supported empirically e.g., [50]), group cohesion can be founded on what is called *organic solidarity*, where individuals are valued for their own distinct strengths or roles and feel essential to the collective effort. One participant vividly illustrated this, noting that their leader’s check-ins, exercise adaptations, and personal touches made her feel “very warm” and like she “wanted to go to the next session.” These insights invite further exploration of complementarity as a mechanism through which social identity leadership strategies (particularly those grounded in individualisation and inclusion) foster social cohesion and long-term engagement.

Strategies grounded in identity advancement received the most consistent support, suggesting that participants particularly valued leaders who clearly acted in the interests of the group and its members. Strategies reflecting entrepreneurship (e.g., establishing norms) and impresarioship (e.g., creating rituals, visual content, or tools) received more variable ratings. For example, while thematic and symbolic elements that made the class distinctive (e.g., themed sessions and motivational words) were not universally endorsed, for some participants these played a meaningful role in fostering emotional engagement and making the class feel personally relevant. This aligns with emerging research positioning novelty as a core psychological need within self-determination theory (SDT) [51], with evidence linking it to improved motivation, wellbeing, and health behaviours, including in people undergoing cancer treatment [52]. In this context, identity impresarioship strategies may be most effective when they are used to complement foundational practices of care and responsiveness, adding emotional resonance and helping bring the group identity to life.

While most participants valued strategies that fostered interaction and group cohesion, a small subset consistently rated these strategies as less appealing. This divergence warrants attention. Groups need to feel meaningful and valued to foster engagement and the variation in responses in this study reinforces that there is no “one-size fits all” solution that will work equally well for everyone. When selecting strategies, exercise leaders should consider the unique values and preferences of the group members, as well as their own comfort and capacity, to ensure their leadership communicates authenticity and commitment (i.e., “they want to be there”). In cancer settings, some participants may prefer to exercise independently [53], yet may only have free access to expert support through group-based classes. Offering flexible delivery models that accommodate different forms of participation (e.g., recording an exercise video to support independent exercise) may help to improve a sense of belonging to the group while respecting their need for autonomy.

The participant-identified strategies we derived also reflect principles embedded in identity leadership training, specifically the 5R program [54]. This structured approach walks leaders through five key stages—Readying, Reflecting, Representing, Realizing, and Reporting—with a focus on managing group identities and enhancing group cohesion and effectiveness. Notably, the Reflecting stage involves defining shared group values, which is apparent in our highest-rated social support strategy of setting respectful group rules. Similarly, the Representing stage centres on identifying shared aspirations, which aligns closely with participants’ strong endorsement of collaborative goal-setting and monitoring strategies. These overlaps highlight the translational potential of the SIGN framework: participants, drawing solely on lived experience, identified strategies consistent with theoretically grounded leadership development programs. This suggests that SIGN may provide not only a consumer-informed framework but also a practice-friendly entry point for implementing social identity leadership in exercise oncology and broader supportive care settings.

From a policy and systems perspective, these findings have important implications for advancing equitable and culturally safe cancer support services, as emphasised in the Australian Cancer Plan [55]. For priority populations, including Aboriginal and Torres Strait Islander peoples, LGBTIQ + individuals, those from culturally diverse backgrounds, and people in rural or remote areas, strategies that are culturally and contextually relevant may play a key role in fostering a sense of shared identity and belonging. While our sample did not include sufficient representation from these groups to explore this in depth, future research should investigate how intersecting identities might shape experiences of leadership, inclusion, and group cohesion in supportive care settings. It is also possible that identity prototypicality, the extent to which leaders are seen as representative of the group, may carry greater relevance among marginalised communities, where shared lived experience can enhance trust, safety, and perceived legitimacy. This is consistent with scholarship highlighting the role of lived experience in fostering credibility and countering epistemic injustice, particularly for those whose voices have historically been excluded or devalued [56].

### Strengths and limitations

This is the first study to apply identity leadership theory within a cancer survivorship context, offering a novel theoretical contribution to the supportive care literature. A key strength is the participatory design, which centred lived experience and generated strategies that are both theoretically grounded and pragmatically useful. The SIGN framework reflects core behaviour change principles while uniquely incorporating mechanisms that support identity development and group cohesion.

However, the study has several limitations. This study focuses specifically on telehealth group classes. Application to other digital delivery modes is unclear. Participants were generally physically active and highly educated, and the study focused specifically on individuals with a lived experience of blood cancer. Although we captured the perspectives of regional cancer survivors (approximately 60% of the sample), representation from other priority populations was limited. These factors reduce the generalisability of the findings, particularly for people with other cancer types and to those who are less physically active or who face greater socio-economic or cultural barriers. Additionally, while the strategies were co-developed based on lived experience, their implementation and impact were not evaluated in real-world settings, meaning their feasibility and effectiveness remain to be tested.

### Future research

Future research should assess the utility of the SIGN framework across a range of delivery modes (e.g., face-to-face, hybrid, digital), clinical populations, and professional settings. This includes evaluating whether training exercise professionals in identity leadership and the use of SIGN enhances participant engagement, satisfaction, and health outcomes. Future research may also consider the potential for “digital social identity leaders”. For example, with a self-management app, researchers can examine how features like prompts, automated feedback, and goal-setting could be designed to actively cultivate shared social identities. The findings may also have direct relevance for emerging digital health tools, such as AI-chat bots, where digital “leaders” could be trained to deliver SIGN-based support. Regardless of delivery format, to support equitable care, further investigation is needed into how intersecting identities shape individuals’ experiences of leadership, inclusion, and group cohesion, particularly among marginalised populations who may face additional barriers to participation. The 29 strategies are not proposed as a definitive list, but rather as a preliminary starting point that is consumer-informed and theory-based. SIGN itself should be iteratively refined and improved as needed.

A key direction for future work is understanding how to support engagement among individuals who do not experience a strong sense of group fit. Flexible delivery models that offer both structured group formats and self-guided options may help accommodate diverse needs and preferences, particularly when paired with leadership approaches that support autonomy and connection. Additionally, there is significant potential for technology to play a role in fostering social identification, for example, by incorporating shared symbols, routines, or narratives into digital platforms. Designing digital environments that strengthen group identity and psychological safety represents a promising and underexplored avenue for enhancing motivation and connection in supportive care programs.

### Conclusion

This study identified 29 consumer-informed strategies that can support engagement in online exercise programs for cancer survivors. These strategies, mapped to social identity leadership theory, were synthesised into the SIGN framework, a practical tool that integrates behaviour change techniques with identity-supportive practices. The framework encourages leaders to promote engagement not just through personalised care, but by fostering connection, group cohesion, and shared purpose. By bridging theory and lived experience, SIGN offers a promising foundation for enhancing motivation and inclusion in cancer exercise programs and broader supportive care.

## Supplementary Information

Below is the link to the electronic supplementary material.

ESM 1 (DOCX 17.0 KB)

ESM 2 (DOCX  30.0 KB)

## Data Availability

De-identified data is available from the corresponding author on reasonable request.
