# Supplementary material for: Co-designing strategies to enhance engagement in telehealth exercise classes among cancer survivors: a participatory study applying identity leadership theory and introducing the SIGN framework
Source: J Cancer Surviv. 2026 Feb 6;20(Suppl 2):1489–502. doi: 10.1007/s11764-026-01966-x (PMC13744552; doi:10.1007/s11764-026-01966-x)
Supplement: Supplementary file 1 [file 11764_2026_1966_MOESM1_ESM.docx]

Supplementary Table 1. Participant characteristics

|  | N = 18 |
| --- | --- |
|  | Mean (SD) |
| Age (years) | 61.61 (7.7) |
| BMI (kg/m2) | 27.24 (6.07) |
| Gender | N (%) |
| Male | 8 (44.44) |
| Female | 10 (55.56) |
| Cancer Type | N (%) |
| Leukaemia | 4 (22.22) |
| Myeloma | 11 (61.11) |
| Lymphoma | 2 (11.11) |
| Other | 1 (5.56) |
| Disease Status | N (%) |
| I have completed treatment and am in remission | 8 (44.44) |
| I have ongoing treatment to manage the disease | 8 (44.44) |
| I am currently undergoing curative treatment | 2 (11.12) |
| Relationship Status | N (%) |
| Married, de-facto or living with a partner | 16 (89) |
| Separated or divorced | 1 (5.55) |
| Single | 1 (5.55) |
| Education | N (%) |
| University degree | 11 (61.11) |
| Certificate or diploma (e.g., TAFE or college) | 3 (16.67) |
| High school (Year 10 or 12) | 3 (16.67) |
| Other | 1 (5.55) |
| Employment | N (%) |
| Full-time/part-time/casual | 8 (44.44) |
| Retired | 5 (27.78) |
| Other | 5 (27.78) |
| Location | N (%) |
| Major City | 7 (38.89) |
| Regional | 11 (61.11) |
| Physical Activity Status^+^ | N (%) |
| Active (≥24) | 16 (89) |
| Moderately Active (14-23) | 1 (5.55) |
| Insufficiently Active/Sedentary (<14) | 1 (5.55) |

^+^Assessed using the Godin Leisure Time Exercise Questionnaire
