# Supplementary material for: Co-designing strategies to enhance engagement in telehealth exercise classes among cancer survivors: a participatory study applying identity leadership theory and introducing the SIGN framework
Source: J Cancer Surviv. 2026 Feb 6;20(Suppl 2):1489–502. doi: 10.1007/s11764-026-01966-x (PMC13744552; doi:10.1007/s11764-026-01966-x)
Supplement: Supplementary file 2 [file 11764_2026_1966_MOESM2_ESM.docx]

**Supplementary File 2:**

Supplementary Table 2: Initial deductive coding of consumer-identified strategies, showing which strategies were mapped to which Identity Leadership Constructs.

|  |  |  | On a scale from 0 (not at all) to 100 (to a great extent), please rate how much you agree that the following strategies would help you **feel committed to the online exercise group.** | | | | | | | On a scale from 0 (not at all) to 100 (to a great extent), please rate how much you agree that  the following strategies would **appeal to you.** | | | | | |
| --- | --- | --- | --- | --- | --- | --- | --- | --- | --- | --- | --- | --- | --- | --- | --- |
|  |  | SIGN | Mean | SD | Median | Min | | | Max | Mean | SD | Median | | Min | Max |
|  | Identity Entrepreneurship | | | | | | |  | | | | | | | |
| 1 | Build trust within the group to make people feel comfortable sharing their struggles or concerns towards doing the exercise (e.g., no judging) | Social connection and support | 78.1 | 26.7 | 84.0 | 14.0 | | | 100.0 | 69.5 | 28.1 | 80.0 | | 5.0 | 100.0 |
| 2 | Set up clear and respectful group rules (e.g., taboo topics - particular topics that shouldn't be discussed within the group) | Social connection and support | 77.6 | 30.0 | 90.5 | 10.0 | | | 100.0 | 76.1 | 30.4 | 84.0 | | 10.0 | 100.0 |
| 3 | Make time to talk during the class, allowing people to share their stories, thoughts, concerns, and experiences | Social connection and support | 69.5 | 25.7 | 70.5 | 20.0 | | | 100.0 | 69.3 | 28.1 | 79.0 | | 9.0 | 100.0 |
| 4 | Refer to the group using collective language (e.g., "the team", "we", and "us") | Social connection and support / Inclusion | 60.3 | 29.3 | 62.5 | 10.0 | | | 100.0 | 55.6 | 37.3 | 60.0 | | 6.0 | 100.0 |
| 5 | Highlight the group's shared goal of achieving physical fitness, improved wellbeing, and a better quality of life | Goals | 69.7 | 31.6 | 79.0 | 5.0 | | | 100.0 | 76.0 | 27.1 | 84.5 | | 11.0 | 100.0 |
| 6 | Come up with a group name for the class reflecting on the group aim (i.e., what the class is for) | Novelty | 60.0 | 28.5 | 62.0 | 10.0 | | | 100.0 | 57.8 | 37.5 | 58.0 | | 7.0 | 100.0 |
|  | Impresarioship | | | | | | |  | | | | | | | |
| 1 | Create opportunity for people to interact outside of the class (e.g., discussion board, emails communication) | Social connection and support | 69.9 | 28.5 | 75.0 | 6.0 | | | 100.0 | 64.2 | 32.5 | 68.5 | | 4.0 | 100.0 |
| 2 | Organize a group chat on social media (e.g., a Facebook group) | Social connection and support | 64.8 | 29.6 | 74.5 | 4.0 | | | 100.0 | 64.3 | 33.2 | 71.5 | | 4.0 | 100.0 |
| 3 | Facilitate options to hang around immediately before or after the class (e.g., stay behind on zoom) | Social connection and support | 63.4 | 30.4 | 72.5 | 5.0 | | | 100.0 | 63.3 | 34.5 | 69.5 | | 4.0 | 100.0 |
| 4 | Ask the group to share their individual short-term and/or long-term goals | Goals | 71.2 | 30.0 | 81.5 | 1.0 | | | 100.0 | 74.4 | 27.4 | 84.5 | | 10.0 | 100.0 |
| 5 | Implement a structured tracking system (e.g., a scoreboard, spreadsheet, diagram) to measure individual and group progress | Goals | 67.9 | 24.5 | 71.0 | 25.0 | | | 100.0 | 73.4 | 25.7 | 81.0 | | 15.0 | 100.0 |
| 6 | Introduce weekly themes based on groups interests (e.g., grand-final week, managing fatigue) | Novelty | 65.9 | 29.2 | 71.0 | 10.0 | | | 100.0 | 58.2 | 31.1 | 59.0 | | 1.0 | 100.0 |
| 7 | Introduce motivational word of the week for the group to reflect on (e.g., strength, hope, peace, mindfulness) | Novelty | 62.9 | 30.9 | 65.5 | | 1.0 | | 100.0 | 64.3 | 32.5 | | 72.5 | 1.0 | 100.0 |
| 8 | Involve the group in music selection for class warm-ups and cool-downs | Novelty | 58.7 | 24.3 | 61.0 | 18.0 | | | 93.0 | 54.5 | 29.3 | 56.0 | | 9.0 | 100.0 |
| 9 | Introduce weekly challenges (e.g., take a photo of a bird while out for a walk) | Novelty | 57.6 | 29.5 | 56.5 | 1.0 | | | 95.0 | 60.8 | 29.5 | 64.0 | | 1.0 | 100.0 |
| 10 | Put up slides or content centred around the group that are visually relevant to the group aim or name | Novelty | 55.1 | 31.9 | 60.5 | 4.0 | | | 100.0 | 54.7 | 33.6 | 59.0 | | 1.0 | 100.0 |
|  | **Advancement** | | | | | | |  | | | | | | | |
| 1 | Recognize people's commitment to the class and provide group support as an encouragement for that commitment (e.g., cheer for each other’s accomplishments) | Social support and connection | 67.5 | 25.0 | 73.0 | 19.0 | | | 100.0 | 64.6 | 32.4 | 72.0 | | 5.0 | 100.0 |
| 2 | Work with the group to identify their aims/goals as a group | Goals | 70.5 | 28.0 | 82.5 | 15.0 | | | 100.0 | 65.8 | 32.9 | 75.5 | | 7.0 | 100.0 |
| 3 | Put together a video for the group detailing the exercise and show how to do the exercise correctly | Novelty | 87.7 | 19.5 | 92.5 | 22.0 | | | 100.0 | 81.3 | 26.0 | 87.0 | | 12.0 | 100.0 |
| 4 | Structure the exercise to match with individual's needs and physical circumstances | Individualisation | 91.5 | 9.2 | 95.0 | 75.0 | | | 100.0 | 88.2 | 15.2 | 94.0 | | 55.0 | 100.0 |
| 5 | Design the program based on group needs (e.g., always having a go slow option or easier alternative for each exercise) | Individualisation | 89.3 | 12.2 | 93.0 | 60.0 | | | 100.0 | 85.7 | 14.5 | 89.5 | | 55.0 | 100.0 |
| 6 | Deliver the class in short exercise segments, allowing people to have breaks and/or a shorter session if needed | Individualisation | 74.7 | 20.4 | 82.5 | 34.0 | | | 100.0 | 75.3 | 27.5 | 85.0 | | 10.0 | 100.0 |
| 7 | Do a survey prior to draw on the group's common grounds, understand their needs and interests | Individualisation | 70.9 | 23.7 | 78.5 | 15.0 | | | 100.0 | 68.4 | 29.1 | 76.0 | | 10.0 | 100.0 |
| 8 | Set up personalized goals for individuals based on each person's physical ability, fitness, and/or treatment level | Individualisation | 89.3 | 16 | 99.5 | 56 | | | 100 | 91.9 | 12.2 | 97.5 | | 62 | 100 |
|  | Not a clear example of identity leadership |  |  |  |  |  | | |  |  |  |  | |  |  |
| 1 | Pair up people with similar fitness, treatment level and/or interests (e.g., buddy system) | Social connection and support | 73.3 | 22.0 | 77.5 | 20.0 | | | 100.0 | 74.5 | 29.3 | 88.0 | | 11.0 | 100.0 |
| QUOTE: Form could form ease with people at similar stage of treatment, although no two treatments are identical, but you could discuss where you are at and motivate each other if you sort of form groups within groups. It isn’t just the leader motivating you but others in the group **(ID10, Male, 68 years).** I didn’t know anyone who had a bone marrow transplant it was completely foreign to me and new, so being in this group is great (ID1, Female, 67 years).  ACTION: recoded as Advancement | | | | | | | | | | | | | | | |
| 2 | Conduct baseline and follow-up assessments (e.g., physical ability testing) | Individualisation | 88.2 | 15.5 | 97.0 | 51.0 | | | 100.0 | 82.5 | 22.7 | 90.5 | | 26.0 | 100.0 |
| QUOTE: And I guess it’s also useful to have a measurement so that you can do, you start off with a small amount and then you work up to a larger amount and it shows you that you are improving (**ID3, Female, 56 years)**; You need to work out what level the class is at in terms of the activities that are being conducted… Sometimes people overrate their abilities and so if you are leading that class you have to understand exactly where they are at in terms of what you are trying to impart to them (**ID 10, Male 68 years**). ACTION: recoded as Advancement | | | | | | | | | | | | | | | |
| 3 | Get to know how people are feeling on a particular day (e.g., traffic light system: red/yellow/green – well/soso/unwell) and adapt the exercise based on that | Individualisation | 84.4 | 16.3 | 88.5 | 50.0 | | | 100.0 | 81.9 | 24.3 | 89.0 | | 10.0 | 100.0 |
| QUOTE: *At the start of the class, if the instructor asks if anyone has any injuries or anything, and they know everyone’s name, then they modify they call out to the person when they do something, they say look [name] make sure you don’t lift your arm up above your shoulder, cause they know that you’ve got an injury and so it is very directed and caring about particular people in the class.* (Nicole). (**ID18, Female 51 years).**  ACTION: recoded as Advancement/ Entrepreneurship | | | | | | | | | | | | | | | |
| 4 | Get to know people in the group individually (e.g., their limitations, needs) before starting to work with them | Individualisation | 83.5 | 17.4 | 91.5 | 51.0 | | | 100.0 | 83.1 | 14.6 | 84.5 | | 59.0 | 100.0 |
| QUOTE: “*One of the things that I think is really important is actually getting to know people before you start to work with them… so getting to know them individually, getting to know their limitations, what their needs are, and how best you can support them … and I think that sort of engenders that “I’m important, I’m being listened to here, I’m being understood, and I think that can really emanate it into the whole group, whereby the leader is able to cater to the individual needs of people but also integrate those needs within the group so that they feel they are not just an individual but that they feel like they belong.* ***(ID4, Female, 68 years).”***  ACTION: recoded as Advancement | | | | | | | | | | | | | | | |
| 5 | Monitor individual and group progress closely and step in when needed to give feedback | Individualisation, Goals | 83.0 | 16.9 | 84.0 | 49.0 | | | 100.0 | 85.3 | 15.5 | 89.0 | | 43.0 | 100.0 |
| QUOTE: *When you talk about online exercise I’m thinking about it as being a one-way traffic, when in fact as we’ve discussed it goes both ways so the teacher or leader should be giving feedback on an individual basis where necessary. If somebody’s doing it perfectly correctly then they’re good and they should be told that too, they should be given positive feedback and if they need to change the way they’re doing it then that’s part of the leader’s responsibility in ensuring that there’s benefit to be had.* **(ID10, Male, 68 years)**  ACTION: recoded as Advancement | | | | | | | | | | | | | | | |

Note: Five strategies were originally coded as “not a clear example of identity leadership”. The table displays quotes that were reviewed in relation to these strategies, and recoding decisions that were made.
